# Unique Features of Hepatitis B Virus-Related Hepatocellular Carcinoma in Pathogenesis and Clinical Significance

**DOI:** 10.3390/cancers13102454

**Published:** 2021-05-18

**Authors:** Sheng-Han Wang, Shiou-Hwei Yeh, Pei-Jer Chen

**Affiliations:** 1 Hepatitis Research Center, National Taiwan University Hospital, Taipei 10002, Taiwan; f93445114@gmail.com; 2 Graduate Institute of Microbiology, National Taiwan University College of Medicine, Taipei 10051, Taiwan; shyeh@ntu.edu.tw; 3 NTU Center of Genomic and Precision Medicine, National Taiwan University College of Medicine, Taipei 10055, Taiwan; 4 Graduate Institute of Clinical Medicine, National Taiwan University College of Medicine, Taipei 10002, Taiwan; 5 Department of Internal Medicine, National Taiwan University Hospital, Taipei 10002, Taiwan

**Keywords:** hepatitis B virus (HBV), hepatocellular carcinoma (HCC), inflammation, sex hormone, virus integration, circulating tumor DNA

## Abstract

**Simple Summary:**

Hepatitis B virus (HBV) infection is the major risk factor for hepatocellular carcinoma (HCC). Understanding the unique features for HBV-induced HCC can shed new light on the unmet needs in its early diagnosis and effective therapy. During decades of chronic hepatitis B, hepatocytes undergoing repeated damage and regeneration accumulate genetic changes predisposing to HCC development. In addition to traditional mutations in viral and cellular oncogenes, HBV integration into the cell chromosomes is an alternative genetic change contributing to hepatocarcinogenesis. A striking male dominance in HBV-related HCC further highlights an interaction between androgen sex hormone and viral factors, which contributes to the gender difference via stimulating viral replication and activation of oncogenes preferentially in male patients. Meanwhile, a novel circulating tumor biomarker generated by HBV integration shows great potential for the early diagnosis of HCC. These unique HBV-induced hepatocarcinogenic mechanisms provide new insights for the future development of superior diagnosis and treatment strategies.

**Abstract:**

Hepatitis B virus (HBV) infection is one of the important risk factors for hepatocellular carcinoma (HCC) worldwide, accounting for around 50% of cases. Chronic hepatitis B infection generates an inflammatory microenvironment, in which hepatocytes undergoing repeated cycles of damage and regeneration accumulate genetic mutations predisposing them to cancer. A striking male dominance in HBV-related HCC highlights the influence of sex hormones which interact with viral factors to influence carcinogenesis. HBV is also considered an oncogenic virus since its X and surface mutant proteins showed tumorigenic activity in mouse models. The other unique mechanism is the insertional mutagenesis by integration of HBV genome into hepatocyte chromosomes to activate oncogenes. HCC survival largely depends on tumor stages at diagnosis and effective treatment. However, early diagnosis by the conventional protein biomarkers achieves limited success. A new biomarker, the circulating virus–host chimera DNA from HBV integration sites in HCC, provides a liquid biopsy approach for monitoring the tumor load in the majority of HBV–HCC patients. To maximize the efficacy of new immunotherapies or molecular target therapies, it requires better classification of HCC based on the tumor microenvironment and specific carcinogenic pathways. An in-depth study may benefit both the diagnosis and treatment of HBV-related HCC.

## 1. Introduction

Hepatocellular carcinoma (HCC), accounting for 90% of primary liver cancer cases, is one of the common malignancies worldwide since the incidence ranks sixth, and the mortality rate ranks third among all cancers. Due to the lack of specific disease symptoms and reliable diagnostic markers at early stages, HCC is still considered a challenging public health issue. Most HCC cases are usually diagnosed in an advanced stage, and this generally restricts the efficacy of therapies. Compared with other gastrointestinal tract tumors, the prognosis of HCC patients is relatively poor since the 5-year survival rate is less than 20% [1]. Among all etiologic factors attributing to HCC development, chronic hepatitis B virus (HBV) infection is the most important risk factor, which accounts for around 50% of HCC cases overall [2].

The discovery of HBV around 50 years ago leads an intensive investigation of HBV virology, immunology, and pathogenesis, which also lay down the base for the development of an effective vaccine. The implementation of HBV vaccination reduces 90% of chronic hepatitis B (CHB) prevalence in the vaccinated cohorts, as shown in Taiwan and other Asia countries [3,4,5], which results in a parallel decline of young-age HCC [6,7]. Despite the universal vaccination program being implemented for 35 years, there are still about 257 million CHB carriers by WHO estimation [8].

HBV infection stimulates hepatocarcinogenesis via multiple routes, especially through inducing persistent chronic inflammation [9]. About 15% to 40% of CHB patients eventually proceed into end-stage liver diseases, including cirrhosis and HCC [10]. Clinical use of antiviral nucleotide and nucleot(s)tide analogs (NUCs) at the first line efficiently represses HBV replication and reduces inflammation [11,12]. Subsequently, the HCC risk in these NUCs-treated patients is significantly lowered [13,14]. Therefore, long-term antiviral therapies by NUCs become the standard of care for CHB. However, the residual HCC risk in these treated patients is not negligible since the five-year cumulative incidence of HCC in the noncirrhotic population remains up to 6.9%, which is still above the threshold of surveillance [14,15]. One of the reasons is probably due to the unique feature of HBV DNA integration that happens during early infection periods in the majority of HBV-related HCC [16]. The event of HBV integration is not a mandatory process during the HBV life cycle that proceeds through the episomal, covalently linked close-circular DNA (cccDNA). Therefore, the incidental HBV integration into host chromosomes occurs in only 0.1% of infected hepatocytes [17,18], but it is present in 90% of HBV-related HCC [19]. The dominance of HBV integration in the HBV-related HCC strongly implicates its carcinogenic potential.

Since only about a quarter of CHB patients will succumb to HCC, numerous studies have investigated the risk factors from virus and host for HCC development. The viral risk factors include higher viral titer, HBeAg-positive status, specific viral genotypes, and genetic mutations in viral genomes [9]. The host factors contain older age, male gender, familial history, and genetic variants of immune response genes [20]. Among these risk factors, the male gender has been noted as a striking feature in HBV-related HCC. Slight male gender preference is noted in nonviral HCC patients, which, however, is more evident for the HCC cases in chronic hepatitis B (CHB) pandemic areas [21]. The male-to-female ratio ranges from 5:1 to 7:1 for HBV-related HCC but only around 2.8:1 for hepatitis C virus-related HCC [22]. It is noteworthy that this gender difference in the carcinogenic process is progressively increasing during disease evolution. An epidemiological study in Taiwan pointed out that in the asymptomatic carriers, the male-to-female ratio was about 1.2:1, then was increased to 6.3:1 in patients at CHB stage, and finally became 9.8:1 in HCC patients [23]. In a large cohort community-based case–control study in Taiwan, the male CHB patients carried higher HBV viral loads than their female counterparts [24]. Even in the vaccinated cohort, which has been followed up for over 18 years, the prevalence of occult HBV infection was higher in males than in females (10.7% vs. 4.4%) [25]. Therefore, the male gender is believed to be a major risk factor for HBV-related hepatocarcinogenesis, starting from the relatively early chronic hepatitis stage and retained until the late stage of HCC development.

This review article focuses on the unique HBV-induced hepatocarcinogenic mechanisms in detail, covering both male gender and HBV integration, which may provide new insights for medical development in the related fields of pathogenesis, diagnosis, and treatment.

## 2. Pathogenesis of HBV-Related HCC

In the large cohort study from Taiwan, a higher HBV titer in CHB patients is documented to be associated with a higher risk of HCC development [26]. CHB contributes to hepatic malignancy via direct and indirect routes. During the long-term infection process, the HBV-induced persistent inflammation leads to repeated liver damage and hepatocyte regeneration, which help to accumulate tumorigenic mutations, either for growth advantage or immune escape activity, in the hepatocyte chromosomes. Meanwhile, the inflammatory microenvironment provides various signals for stimulating the clonal expansion of hepatocytes containing tumorigenic mutations. In addition to the indirect mechanism, HBV can contribute to the carcinogenic process through direct mechanisms. Integration of HBV genome into the host chromosome may induce the activation of oncogenes by insertional mutagenesis or by disrupting genomic stability. Additionally, the viral proteins such as HBx and PreS deletion mutants could dysregulate cellular signaling pathways involved in promoting hepatocarcinogenesis.

### 2.1. Chronic Inflammation and Hepatocyte Regeneration

In contrast with acute inflammatory responses to acute infection in a short-term period, chronic inflammation against persistent infection is an unwilled process passively setting up a tumorigenic microenvironment as one of the malignant hallmarks [27]. Chronic HBV carriers continuously suffer from repeated episodes of chronic hepatitis for decades, with a 20–30-fold higher risk for HCC development if no intervention therapies are implemented [28]. By contrast, the NUCs-treated patients who achieve sustained viral suppression and declined liver inflammation have reduced or delayed HCC occurrence [29,30]. This thus validates that the persistent hepatic inflammation during repeated HBV infection processes plays an important role in promoting HCC formation.

Complete eradication of HBV infection generally requires the coordinated combination of host adaptive immunity in both cytolytic and noncytolytic routes. This process includes the direct elimination of infected cells by specific CD8+ T-cell-mediated cytotoxicity, secretion of neutralizing antibodies against HBV surface antigen (HBsAg) by B-cells, and assistance of CD4+ T-cells for supporting effective clearance [31]. However, in patients bearing long-term HBV infection, the population of virus-specific CD8+ T-cells is often insufficient or their activities are exhausted. This ineffective but damaging immune response is partly due to the participation of immunosuppressive mediators, such as the interleukin-10 secreted by liver-resident Kupffer cells [32,33]. The events might reduce the influx of circulating HBV-specific T cells into the liver to clear the infected hepatocytes [34,35,36,37]. In this microenvironment, antigen-specific CD8+ T-cells are usually exhausted and unable to eradicate HBV infection. In combination with the stimulation by cytokines and growth factors, sustained hepatic inflammation results in repetitive cycles of liver damage and hepatocyte regeneration. Hence, these CHB-related scenarios set up a liver microenvironment that predisposes the carcinogenic process, from clonal expansion to HCC [38,39,40,41,42].

### 2.2. HBV Genotypes and Specific HBV Variants

Thus far, there are 10 genotypes of HBV, genotypes A to J, distributed with specific geographic areas [43]. Different HBV genotypes appear to associate with distinct biology in the infected populations. For example, in Asia, infection of genotype C of HBV is associated with more severe course of liver diseases such as cirrhosis and HCC; in other pandemic areas, genotype C, D, and F of HBV were reported associated with a higher risk of HCC [44,45]. In addition, the HBV basal core promoter (BCP) mutations at 1762/1764, more frequently identified in genotype C than in genotype B strains of HBV, are significantly associated with the risk of HCC development [46,47,48]. Other than BCP mutations, ample evidence indicates that some naturally occurring HBV variations are also associated with an elevated risk of HCC [43,49].

One category of the naturally occurred HBV mutants, which expresses deleted or mutated HBV surface (HBs) proteins, is correlated with a higher HCC risk. Such mutants appear in CHB patients, ranged from 6% at early infection to 35% at the late stage, and then achieves 60% in the HCC-bearing patients [50]. These mutated HBs proteins have been shown to be potentially oncogenic in transgenic mice and able to induce dysplasia nodules [50,51,52,53,54,55]. The misfolding PreS/S variant proteins accumulated in the endoplasmic reticulum (ER) lumen could initiate the ER stress-induced response for evoking oxidative DNA damage and genomic instability [50]. Alternatively, in an ER stress-independent way, the PreS2 mutant could trigger the decay of p27 and RB or enhance the expression of cyclooxygenase-2 and cyclin A, which also contributes to the oncogenic process [56,57,58]. Despite these observations, the mechanisms of how HBV genotypes or other natural variants modify viral carcinogenesis remain elusive.

### 2.3. HBx: A Multifunctional Viral Protein with Versatile Oncogenic Activities

HBx is essentially required for active HBV replication in vivo [59] but has long been considered as the key viral factor contributing to HBV-induced carcinogenesis, through versatile mechanisms [9]. Expression of sufficient HBx transgene in mouse livers induces orthotopic HCC [60,61,62,63]. Accumulating lines of evidence demonstrate that HBx dysregulates the expression of plenty of cellular genes and activates many signaling pathways in hepatocytes, which dominates the transcriptional control, cell-cycle dynamics, and balance of hepatocyte proliferation and apoptosis [9,64]. These malicious HBx functions drive the infected hepatocytes into an actively proliferating status, which favors HBV propagation but may attribute to cell transformation with pro-oncogenic activity.

#### 2.3.1. HBx Stimulates HBV Gene Expression

For supporting the viral life cycle, HBx is an authentic transactivator for HBV gene expression [65,66], possibly through the decay of the structural maintenance of chromosome 5/6 complex (Smc5/6) [67,68,69]. This heterodimeric complex directly binds the host DNA for sustaining cell chromosome stability and dynamics [70,71,72,73] but topologically entraps episomal HBV cccDNA genomes and restrains viral transcription as host restriction factors [74]. HBx overcomes this host defensive barrier by bridging Smc5/6 with the host E3 ubiquitin ligase, which promotes the degradation of Smc5/6 and thus relieves the restraints on HBV transcription. Through this mechanism, HBx stimulates viral gene expression and replication, as demonstrated in cultured cells and in chimeric mice with humanized hepatocytes [67,68]. Therefore, it could elevate HBV viral loads and exacerbate the CHB-induced inflammation and carcinogenesis.

#### 2.3.2. HBx Effects on Tumor-Related Characteristics

In addition to promote Smc5/6 decay for stimulating viral gene expression, HBx is a multifunctional regulator in modulating a variety of tumor-related cellular pathways and biological functions. One well-demonstrated function is that HBx can aberrantly activate β-catenin pathway activity in hepatocytes, which is essential for their self-renewal and regeneration activity in the chronic inflammatory livers [75,76,77]. As noted, HBx expression is closely associated with activation of β-catenin in up to 80% of HBV-related HCC [78]. In the mouse model with β-catenin conditionally knockout in matured hepatocytes, which spontaneously develops the senescence-associated chronic hepatitis at old age, expression of HBx accelerates the replacement of senescent hepatocytes by stimulating the growth of hepatic progenitor cells (HPCs) in the periportal area. HBx could amplify the complement C1q-mediated activation of β-catenin pathway and thus carcinogenic potentials in the expanding HPCs. This mechanism highlights the oncogenic potential of HBx in the β-catenin-mediated HPC regeneration in the inflammatory liver and has been validated in clinical specimens [79,80].

Moreover, accumulating lines of evidence have pointed out that HBx could promote hepatocyte transformation through interacting with a variety of cellular proteins, which modulate the DNA repair process, autophagy function, cell proliferation, and some other tumor-related activities [81,82]. However, more evidence from in vivo models and clinical cohorts is demanded to validate these versatile oncogenic activities of HBx further.

#### 2.3.3. HBx Mutants and HCC Development

Several genetic mutations in HBx have been identified in clinical HCC specimens, which are associated with a higher risk of HBV-related HCC [83]. Some of these HBx mutants were experimentally approved in vitro with a higher capability to enhance HBV replication and promote hepatocyte proliferation when compared with the wild-type HBx [84]. In addition, expression of truncated HBx has been observed in some HCC, which may be implicated in liver carcinogenesis [85,86,87]. However, inactivating alterations of HBx have been identified in more than 70% of HBV-related HCC [88]. These studies might imply that HBx could be involved in the development but not in the maintenance of HCC.

### 2.4. Sex Hormones in Regulating the Gender Difference of Carcinogenesis

One striking characteristic of HBV-related HCC represents its male predominance in different stages of HCC progression, starting from hepatitis to HCC [22,89]. In fact, sex hormones, both androgen and estrogen, have long been considered as important regulators for HBV-related pathogenesis in CHB patients. Previous epidemiological cohort studies identified an association of higher androgen/androgen receptor (AR) pathway activity with a higher risk of HCC in male HBV carriers [90,91]; by contrast, an inverse correlation between estrogen receptor pathway and HCC risk was found in female HBV carriers [92]. This suggests a possible interaction between HBV infection and the androgen or estrogen pathway in regulating the carcinogenic process in HBV carriers with gender.

#### 2.4.1. The HBx–AR Circuit in Promoting Male HCC

Our serial studies did identify an interaction between HBV infection and the androgen pathway, as one carcinogenic mechanism in male HBV-related HCC. HBx is able to enhance the androgen-dependent AR activity, in vitro and in vivo. Through activating c-Src and inhibiting GSK3β kinases, HBx stimulates the phosphorylation of AR and reinforces its dimerization, respectively [93,94,95]. This mechanism promotes the translocation of AR dimers and increment of their transcriptional activities in the nuclei for elevating the expression of AR downstream genes. Interestingly, the active AR could recognize the androgen response elements within the enhancer I of the HBV genome and activates the overall viral transcription, including the HBx gene [96]. It thus drives a positive feedback circuit for the persistent elevation of HBV viral replication and AR pathway activity, preferentially in male HBV carriers [97].

**Figure 1:**
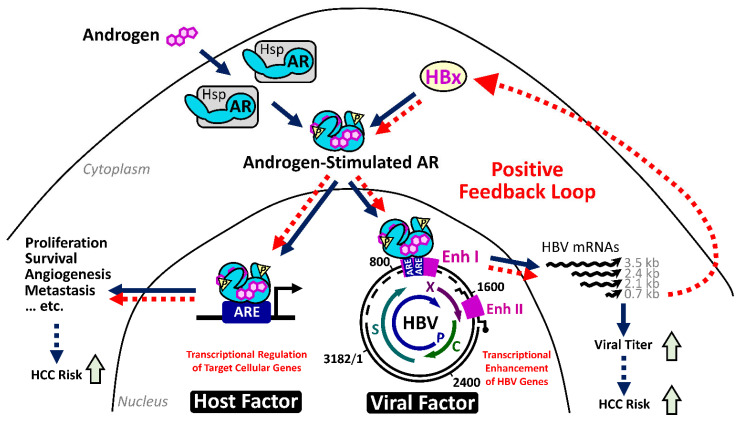
Illustration of the positive feedback loop between HBx and AR for promotion of HBV-induced hepatocarcinogenesis in male CHB patients. In male HBV carriers with chronic infection, HBx enhances the transcriptional activity of androgen-activated AR, which recognizes the androgen-responsive element (ARE) motifs within viral enhancer I (Enh I), thus reinforcing overall HBV gene expression including HBx (the right part). On the other hand, the HBx-activated AR could aberrantly stimulate downstream expression of proto-oncogenes, which facilitate cell proliferation and survival in the carcinogenic process. This positive feedback circuitry may simultaneously elevate serum HBV titer and activate host genes with carcinogenic potentials in infected hepatocytes, leading to the elevated risk of HCC development in male CHB patients.

This circuit increases the viral titer and upregulates the expression of putative AR downstream oncogenic genes in the hepatocytes of male CHB patients (Figure 1). As one example, the HBx-enhanced hepatic AR elevates the expression of miR-216a, which, in turn, represses the expression of tumor suppressor in lung cancer-1 (TSLC1), especially in male HCC cases [98]. Cell cycle-related kinase (CCRK) has been identified as another putative target gene regulated by this HBx–AR circuit, which can stimulate carcinogenesis through upregulation of β-catenin/TCF signaling [95,99].

The carcinogenic potential of the HBx–AR circuit was demonstrated in cell culture and in the animal model [94]. Deprivation of AR pathway activity, by castration or by genetic knockout of hepatic AR expression, significantly reduced the HCC incidence in the HBx transgenic male mice. The results support the critical contribution of AR activity in HBx-induced male HCC [100]. A substantial decrease of alanine aminotransferase in the AR-deprived HBx transgenic mice further suggested a putative function of hepatic AR activity in maintaining the persistent inflammation [100], which is worthy to be investigated in the future.

#### 2.4.2. Estrogen/Estrogen Receptor Pathway in Suppressing Female HCC

In contrast with the proto-oncogenic role of the androgen pathway in male HCC, the estrogen axis displays defensive effects to mitigate the progression of female HCC. The first clues from cohort studies showed that deprivation of estrogen due to menopause or oophorectomy increases the HCC incidence in female HBV carriers [92,101,102]. This hypothesis has been in vivo supported by animal studies. For example, in the diethylnitrosamine-induced HCC mouse model, which reflects the sex disparity of human HCC, the estrogen/estrogen receptor α (ERα) axis alleviates the inflammation-induced liver injury by blockade of interleukin-6 secretion from hepatic Kupffer cells [103]. At the molecular level, the active ERα may behave like an antioxidant to control the reactive oxygen species (ROS)-evoked cell damage via inhibition of NF-κB activation, which downregulates the expression of inflammatory genes dominating the stress responses in persistently injured liver and attenuates cellular ROS burden [104,105,106].

Decreasing the HBV viral replication has been identified as another defensive function of the estrogen pathway in liver carcinogenesis. ERα can suppress the expression of all HBV viral genes by squelching the binding of hepatocyte nuclear factor 4α (HNF4α), an essential transcriptional factor required for HBV mRNA production, to the viral enhancer I. This ERα-mediated restriction passively reduces the viral transcription and thus the viral proteins, including the oncogenic HBx protein, and viral titers in female HBV carriers [107,108]. A decrease of hepatic ERα, which was identified in more than 70% of female HCC, through elevation of miR-18a as one mechanism [109], might abrogate its protective function and contribute to the female hepatocarcinogenesis.

These results altogether demonstrated a mechanism for the opposite effects of androgen and estrogen sex hormones on HBV replication and the HBx-mediated carcinogenic process. It provides an explanation for the increased male susceptibility to HBV-related HCC.

### 2.5. HBV Integration Induced Mutagenesis and Genomic Instability

As an episomal form, the cccDNA of HBV transcribes four major viral transcripts in the viral replication cycle; the longest 3.5-kb transcript is encapsidated into viral capsids for reverse transcription and replication [110]. The newly synthesized HBV genomes that exist as relax circular DNA in most viral nucleocapsids will be enveloped and secreted as matured virions. However, in less than 30% of nucleocapsids, the replicative HBV genomes represent double-stranded linear DNAs due to differential priming in the reverse transcription process. This type of viral genome renders HBV to gain potential access to integrate its DNA into host chromosomes, probably through the noncanonical route of nonhomologous end-joining machinery [111]. Integration of HBV genome may induce insertional mutagenesis and genomic instability.

#### 2.5.1. Random Integration of HBV with Selective Hotspots in HCC Genome

Despite the HBV integration event is not necessary for HBV infection, most HBV-related HCC tumors harbor HBV integrations, which are present in around 90% of cases [16]. This integration event appears to occur at an early phase of viral infection since it could be detected in CHB livers at a very young age [16]. Careful studies indicated that the HBV integration occurs in about 0.1% of acutely infected hepatocytes or in the liver of HBV-infected chimpanzee [17,18,112]. Due to the high prevalence of integration events found in tumors, this indicates a strong advantage of hepatocytes with HBV integration during the selection process of carcinogenesis. Therefore, the role of HBV integration in liver pathogenesis attracts much attention.

As documented, most HBV integrations are randomly distributed across all human chromosomes in the hepatocytes during the chronic hepatitis stage [19,113,114]. However, recent next-generation sequencing (NGS) analysis of HBV-related HCC revealed a few integration hotspots in close proximity of the oncogenes of telomerase reverse transcriptase (TERT) in 25%, mixed-lineage leukemia 4 (MLL4/KMT2B) in 15%, and cyclin E1 (CCNE1) in 5% of HCC cases, respectively [19].

**Figure 2:**
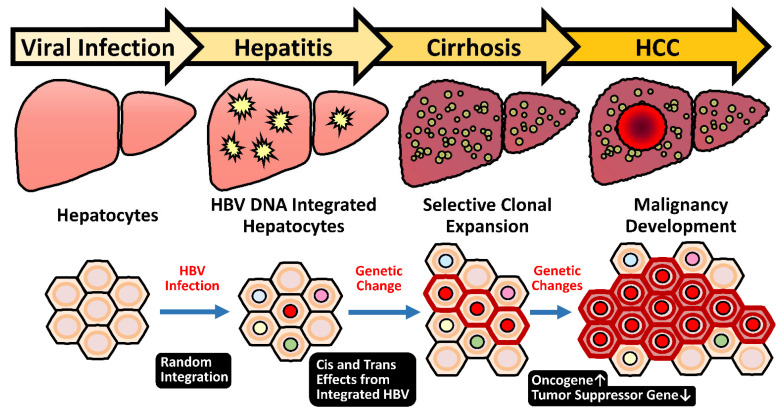
Insertional mutagenesis by integration of HBV genome predisposes potential genetic changes for selective clonal expansion in HCC development. After HBV infection, random integration of viral genome may occur (about 0.1%) in infected hepatocytes. The genetic cis and trans-regulatory effects derived from integrated HBV sequences may render the hepatocytes obtain a growth advantage. From CHB stage to cirrhotic phase, the repeated cycles of cell damage and hepatocyte regeneration may predispose the accumulation of other genetic changes. Therefore, this chronic inflammatory microenvironment sets up a scenario that promotes the hepatocytes with advantageous HBV insertional mutagenesis for clonal expansion and eventually selects for HCC formation.

The hotspots for the breakpoints in the viral genome are clustered at around the direct repeat 1 (DR1) and 2 (DR2) regions, and in most cases, the inserted HBV genome still maintains functional viral enhancers [19,115]. The integrations render the adjacent cellular genes under the transcriptional control of HBV enhance I and thus increase their expression levels in the virus-infected hepatocytes. In the cases that the flanking cellular genes are oncogenic, this event will provide the hepatocytes with a growth advantage for clonal expansion and eventually select for HCC formation. This mechanism belongs to an early carcinogenic event, which starts as early as they could attribute to malignant transformation (Figure 2). Notably, the integration sites are clustered at the promoter regions of TERT and CCNE1 genes, which elevates their mRNA expression levels. The integrations in MLL4, however, are clustered at its introns 3-5, which exert a minor effect on mRNA expression levels [115]. Some other mechanisms for HBV integration to affect MLL4, for example, production of truncated or fusion MLL4 protein, is worth examining. Therefore, the insertional mutagenesis driven by HBV integration in promoter regions or generation of newly oncogenic products might be enriched and selected in long-term carcinogenic process for HCC development. These findings support the HBV integration into these genes favors their evolution to HCC and confirm the insertional mutagenesis hypothesis of HBV integrations.

#### 2.5.2. HBV-Induced Insertional Mutagenesis Is Responsive to Sex Hormone Regulation

In most HBV-related HCC, the HBV genomic enhancer I, which is responsive to the androgen and estrogen pathway, remains intact and functional in the integrated HBV sequence [19,113,115]. It raises a possibility that sex hormones can also target this cis-element for regulating the expression of the flanking cellular genes, similar to the HBV in episomal form. Therefore, the regulations involved in activation of HBV transcription by androgen pathway, but repression by estrogen pathway can also be applied to affect the flanking genes succumbed to insertional mutagenesis mechanisms and thus contribute to the HCC development.

In fact, the capture-based NGS analysis did reveal that the HBV integration at the TERT promoter region occurs more frequently in the male HBV-related HCCs than those in females. The effects of sex hormone pathways on the expression of the TERT gene under the control of integrated HBV at the promoter region have been further confirmed by the reporter assay, which is augmented by AR activity and dependent on HNF4α. Interestingly, another mechanism to activate the TERT expression in HCC, via the -124G > A mutation in the TERT promoter region, was also found to be activated by the androgen pathway [115]. The elevation of the TERT gene by AR pathway through the integrated HBV or specific point mutation at the promoter region thus becomes another molecular mechanism for the male dominance of HBV-related HCCs. The results meanwhile pointed out the TERT and AR as molecular targets for intervention of HBV-related male HCC development.

#### 2.5.3. Genome Instability Caused by HBV Integration

Except for the hotspot genes, the contribution of most HBV integration even to the carcinogenic process remains unclear [19]. Many viral integrations even occur at the genomic regions where they neither disrupt the structure of a gene nor change the level of gene expression and hence are functionally silent. As noted, the insertional mutagenesis has been found to be correlated with the copy number increment at the genetic loci of HBV insertional breakpoints, thus implying the malicious potential of HBV for genomic instability [116]. Through a “hit and run” mutagenesis mechanism, the postintegration rearrangement of cell chromosomes might lead to a wide range of genetic changes within the host genome, including deletions, translocations, production of fusion transcripts, and generalized genomic instability [117,118]. This might be associated with chromosomal deletions, as many of these lost segments contain known tumor suppressor genes such as p53, Rb, cyclin D1 and p16 [119], thus predisposing genetic lesions for the transformation of the hepatocytes bearing with HBV integrations.

### 2.6. Heavy Alcohol Consumption and Risk of HBV-Related Disease Progression

In addition to viral factors, habitual ethanol intake is an independent predictor of death in CHB patients [120]. Light to moderate alcohol consumption (<25 g per day) associates with a 1.5-fold increased risk of HBV-related disease progression, which was only observed in large cohort researches and always not significant in smaller studies. By contrast, alcohol abuse (>60 g/day) obviously accelerates the disease progression to cirrhosis and elevates the risk of HCC incidence about two- to eightfold in CHB patients [121]. Indeed, it had been documented in the experimental model since heavy alcohol intake and HBV synergistically promoted the development of hepatic steatosis in the mice fed with high-fat diet [122]. Moreover, in the meta-analysis, the alcohol consumption in CHB patients raised the relative risk of hepatic steatosis by 43%, compared with those who did not drink alcohol [122]. Ethanol may enhance HBV replication, repress host immune response, and induce oxidative stress, which might set up a fibrotic microenvironment to promote HCC development [123]. However, most of these findings have not been extensively characterized and verified. In addition, the consumption threshold of alcohol for the determination of risky exposure remains uncertain in patients with chronic hepatitis. More studies are needed to delineate further the impact of excess alcohol on liver disease progression in HBV patients.

## 3. Diagnosis of HBV-Related HCC by Circulating Virus–Host Chimera Tumor DNA Generated by HBV Integration

HCC is usually a silent tumor progressing with mild or no symptoms unless tumor burdens large enough or invasion of the vasculature at an advanced stage. The prognosis of HCC depends upon the stage of diagnosis. The curative treatments, such as surgical resection or liver transplantation, can be applied only to the HCC diagnosed at very early or early stages; the 5-year survival rate can reach 50–70% [124]. Unfortunately, about 60% of HCC are diagnosed at the intermediate or advanced and terminal stages by routine tumor surveillance. Even with the advent of recent molecular target or immune-oncological therapies, the HCC response rate improves to 20–30%, but the long-term survival is still disappointed [125]. In addition, those early HCC patients undergoing curative treatment still confront with high HCC recurrence. The recurrence within 1 year after resection significantly shortens the survival of HCC patients [126]. Early diagnosis of HCC tumors and detection of minimal residual lesions after curative treatment still remain as unmet medical needs.

Currently, the diagnosis of HCC mainly relies upon imaging detection and blood protein biomarkers [127,128]. The noninvasive imaging approaches show the sensitivity up to 80% for advanced HCC but less than 50% at detection of early tumors, with a diagnostic limit of 1–2 cm [129,130,131,132,133]. A more applicable serum biomarker, α-fetoprotein (AFP), has high detection specificity of 80–90% but with low sensitivity of 40–65% [128,134]. To improve the diagnostic accuracy of early HBV-related HCC tumors, another serum biomarker, namely, protein induced by vitamin K absence or antagonists-II (PIVKA-II), had been practically evaluated in large cohort studies for combinatorial use with AFP, although the detection sensitivity and specificity for tumors <2 cm was only marginally improved [135,136,137]. Moreover, numerous serum proteins and microRNAs (miRNAs) are recently reported to be potential surrogates for diagnosis of HBV-related HCC, such as deoxyribonuclease 1-like 3 (DNASE1L3), α-L-fucosidase (AFU), γ-glutamyl transferase isoenzyme II (GGT-II), glypican-3 (GPC3), hepatocyte growth factor (HGF) and miR-487b. Individual combination of these novel biomarkers with AFP improved the diagnostic sensitivity of HCC achieved to approximately 90% [138,139,140], depending upon stages. Besides, the integrated bioinformatics analysis based on the HBV-related HCC transcriptome databases suggested the key hub genes with a dysregulated expression profile as potential diagnosis and/or prognosis signatures [141]. However, more prospective lines of evidence from large cohorts and experimental results with solid molecular explanations are needed to support the clinical application of these novel serum biomarkers or genetic tools. Therefore, the requirement of a more sensitive blood tumor marker for small HCC tumors remains a challenge.

Recently, cell-free tumor-specific DNA (ctDNA) has emerged as a new category of circulating biomarker in monitoring tumor progression or resistance to chemotherapies [142,143,144,145]. Nevertheless, for HCC, the detection rate of conventional ctDNA in <5 cm HCC is relatively low, usually <5% [146,147]. One disadvantage for the development of ctDNA marker derives from the rare occurrence of traditional signatures, such as somatic mutations in P53 or β-catenin gene, which are barely applicable for HCC diagnosis due to their limited prevalence (usually <30% in HBV-related HCC) [148]. Another limitation comes from the difficulty in separating these somatic mutation-containing ctDNA fragments from excessive homologous circulating DNAs released from normal cells, which largely interferes with diagnostic specificity.

A novel ctDNA biomarker released from HBV-related HCC tumors was recently proposed to overcome these limitations, namely, the circulating DNA fragment generating from the junctions of HBV integration in the chromosomes of HCC. This virus–host chimera DNA (vh–DNA) consists of the junctional fragments at the HBV integration site, containing both virus and human DNA sequences [149]. Since the randomly distributed HBV DNA integrations have been identified in ~90% of HBV-related HCC [19], the integration-derived vh–DNA can be applied as a biomarker for detecting the majority of HBV–HCC. As the HBV integration is unique to individual HCC tumors, the vh–DNA fragment released in circulation during tumor turnover can be traced as the signature marker for each HCC.

The feasibility of the vh–DNA in detecting HCC has been examined in a cohort of HBV-related HCC patients undergoing tumor resection. The HBV integrations were identified in 88% of the resected HCC by the capture-based NGS platform. For individual HCC, the vh–DNA specific droplet digital PCR assay was established for quantification of the specific vh–DNAs in the plasma collected before and after tumor resection. The vh–DNAs were detected in the baseline samples in 97.7% of the HBV-related HCC patients. At the same time, their detection levels correlated with the tumor sizes (with detection limit at 1.5 cm diameter). By monitoring the residual circulating vh–DNA in the blood collected after surgery, 90% of the patients with detectable vh–DNA levels in 2 months postsurgery experienced HCC recurrence within 1 year [149]. Therefore, the vh–DNA generated by HBV integration might become a new circulating ctDNA biomarker for detecting the tumor load in most HBV-related HCC patients. Furthermore, the unique signature of each vh–DNA are also helpful in monitoring residual tumor and recurrent clonality after tumor resection. Its applicability for detecting primary HCC is worthy of investigation.

In the post-era of applying direct antiviral agents against chronic hepatitis C, the occurrence rate of HCC is substantially decreased below 5% in the treated population [150]. However, in the patients who showed the curative responses but still developed HCC, the pooled estimates in the recurrence and prognostic survival were extremely variable, probably due to the combined heterogenic etiologies [151]. Similar to fighting with HCV-related HCC, the NUC drugs are the major regimens that effectively suppress HBV replication, although the curable outcome is barely achieved. The long-term use of NUCs against CHB significantly reduces the HCC risk in the treated HBV patients [152]. Furthermore, treatment of tenofovir or entecavir in the HBV-related HCC patients after tumor resection obviously decreases the recurrence rate and prolongs overall survival periods [153,154]. Therefore, pharmacological interventions and the programmed surveillance dynamically reshape the guidelines and strategies of clinical diagnosis and management in CHB patients to reduce HCC risk [155].

## 4. The Impact of Emerging COVID-19 Pandemic on CHB Patients

Coinfection or superinfection by other viruses, such as hepatitis D, has been approved to accelerate the progression of liver diseases and increase the risk of HCC among some CHB patients [156]. Recently, the impact of emerging COVID-19 on CHB patients has attracted attention, especially in Asia where there is a large CHB population. According to the limited clinical studies, chronic hepatitis B did not increase the severity of COVID-19 infection outcome. At the same time, coinfection of COVID-19 might induce HBV reactivation, albeit the risk is relatively low [157]. These findings need to be validated further in more studies because hepatic immune stages in response to chronic HBV infection are ambiguous and fluctuated in the patients, which might influence the host immune response to the COVID-19 infection. For cancer patients who are at a higher risk for SARS-CoV-2 infection [158,159], tumor burden is generally believed to be one of the risk factors for the prediction of worse outcomes suffering from COVID-19. The risk of severe symptoms was reported to be obviously higher with a fourfold hazard ratio in the patients receiving anticancer treatments within two weeks before COVID-19 diagnosis than those who did not recently [160]. One of the reasons may probably come from the concomitant immunosuppression under cancer therapies. Despite the reported COVID-19 cases with HCC tumors are underrepresented so far, personal protection and therapeutic decisions should be emphasized to minimize the virus exposure and well-balanced based on the accessibility of medical healthcare, respectively.

In general, regular monitoring and management are recommended to be continued for HBV patients. However, during the COVID-19 pandemic, these routine programs may be shut down due to the diversion of funding and overloading of medical services [161]. Therefore, quantification of serum HBV loads and prediction of HCC risk become the prioritized services to be reorganized in time for HBV patients. Recently, rapid and cheap assays of serum HBV titers from limited blood specimens have been developed by simply applying the loop-mediated isothermal amplification or the fluorescence-based polymerase spiral reaction [162,163]. For surveying the HBV patients with the exacerbation probability, the HCC prediction score focusing on the measurement of hepatic fibrosis was recently evaluated in the HBV cohort. It displayed a superior discriminatory performance for identifying CHB patients at high risk of HCC development [164]. The concept of monitoring the at-risk HBV population by surveillance of the patients with progressive fibrosis via noninvasive approaches but not a liver biopsy is expected for cost-effective prevention of end-stage hepatic diseases. For instance, a combined set of miRNAs (hsa-mir-1225-3p, hsa-mir-1238, hsa-miR-3162-3P, hsa-miR-4721, and hsa-miR-H7) has been assessed as a promising diagnostic model with high accuracy for identifying patients with discriminated fibrotic stages [165]. Moreover, the serum lactate level has recently been validated as a representative surrogate to assess the 6-month mortality of HBV-related decompensated cirrhosis [166]. Undoubtedly, in the post-COVID-19 era, all these novel prognostic tools demand more convincing lines of evidence from large prospective cohorts to support their use in clinic scientifically.

## 5. Treatment of HBV-Related HCC

Clinical management of HCC largely relies on the diagnostic stages and residual liver functions. When HCC is diagnosed at an early stage, curative therapies such as local ablation, surgical resection, or even liver transplantation are effective options [167]. For HCC diagnosed at an advanced stage, molecule-targeted therapy and immuno-oncological therapies become the only regimens. Several multiple kinase inhibitor drugs, such as Sorafenib and Lenvatinib, have been used with limited efficacy [168,169]. More recently, immunotherapy, especially anti-PDL1 in combination with anti-VEGF (avastin) has shown higher response rates (about 30%) and significantly prolongs survival [170]. Despite this, the majority of advanced HCC patients do not respond well to immuno-oncology or multitargeted therapy. Biomarkers to separate responsive versus nonresponsive HCC patients are in urgent need of development. Finally, new drugs specific to HBV-related HCC targets, such as overexpressed telomerase genes or androgen receptor pathways, may provide new avenues for more effective treatment in the future.

## 6. Conclusions

The HBV-related HCC development clinically displays the male predominance in CHB patients. In male HBV carriers, HBx enhances the androgen-activated AR pathway which facilitates viral replication and reinforces downstream gene expression with proto-oncogenic activities. By contrast, the estrogen/ERα axis suppresses HBV gene expression, thus reducing HBx expression and viral titers in female carriers. Therefore, these sex hormone pathways substantially contribute to the gender disparity of HBV-induced hepatocarcinogenesis. In addition, integration of the HBV genome may induce insertional mutagenesis to activate oncogenic gene expression or lead to the instability of host chromosomes, which may dysregulate specific gene expression and predispose them to genetic lesions. HBV integration also provides a new approach to use the circulating virus–host chimera DNA as novel HCC diagnostic tools. It helps to detect minimal residual HCC after curative therapy and could also be used for tracing tumor lineages. Together with an advanced understanding of HBV-induced carcinogenesis, our new knowledge may pave the way for novel and better HCC therapy and diagnosis in the future.
